# Risk of cardiovascular disease and death in patients with breast cancer receiving anthracycline-based therapy: A retrospective cohort study

**DOI:** 10.1371/journal.pone.0335083

**Published:** 2025-12-04

**Authors:** Yu-Cheol Lim, Yoon Jae Lee, In-Hyuk Ha, Ye-Seul Lee

**Affiliations:** 1 Jaseng Spine and Joint Research Institute, Jaseng Medical Foundation, Seoul, Republic of Korea; 2 School of Pharmacy, Sungkyunkwan University, Suwon, Republic of Korea; Apeejay Stya University, INDIA

## Abstract

**Background:**

With the increasing survival rate and overall lifespan of patients with breast cancer, increased mortality due to cardiovascular causes is a notable concern. Anthracyclines, which are commonly used and effective chemotherapeutic agents for breast cancer, are known for their cardiotoxicity. Existing studies vary in their findings owing to heterogeneous factors such as study population and design, underscoring the need for high-quality evidence to comprehensively examine the risk factors for anthracycline-induced cardiotoxicity. This study compared the incidence of cardiovascular disease, all-cause mortality, and their associated risk factors following adjuvant anthracycline therapy as the initial anticancer treatment, utilizing National Health Insurance claims data for a nationwide cohort of patients with breast cancer.

**Methods:**

The study cohort comprised patients initially diagnosed with breast cancer between 2011 and 2013 who underwent mastectomy within 6 months of diagnosis and received anticancer therapy within 6 months of surgery. The primary outcomes were composite cardiovascular events and all-cause mortality during a follow-up period of up to 5 years. Secondary outcomes encompassed specific types of cardiovascular events such as congestive heart failure, heart failure and cardiomyopathy, coronary artery disease and cardiac arrest, and stroke. Incidence rates for these outcomes were presented as incidence rate ratios (IRR), and hazard ratios (HR) for individual risk factors were derived using the Cox proportional hazards model.

**Results:**

The study cohort comprised 9,439 patients with breast cancer in both the anthracycline and non-anthracycline groups. There was no significant association between adjuvant anthracycline therapy and the composite cardiovascular events (adjusted HR 0.911 [95% CI 0.823–1.008]). In contrast, all-cause mortality was higher in the anthracycline group (IRR 2.155 [95% CI 1.892–2.455]; adjusted HR 2.160 [95% CI 1.882–2.480]). Adjuvant trastuzumab was significantly associated with an increased risk of the composite cardiovascular event (adjusted HR 1.256 [1.093–1.443]), although no trend was identified concerning adjuvant trastuzumab status in the subgroup analysis. Advanced age and hypertensive comorbidities were risk factors for all outcomes.

**Conclusion:**

To prevent cardiovascular event onset in patients with breast cancer undergoing anthracycline-based chemotherapy, thorough monitoring is essential, especially in patients of advanced age, those with comorbid hypertension, and those undergoing trastuzumab treatment.

## Background

Breast cancer (BC) is the most common malignancy affecting women worldwide. In recent decades, the development of early diagnostic techniques and neoadjuvant and adjuvant therapies have led to reduced mortality and increased survival of patients with BC [1,2]. With increasing survival and duration of disease, patients with BC are exposed to adjuvant chemotherapy regimens for a relatively long period, and several studies have been conducted to examine the side effects of the chemotherapeutic agents [3,4]. A representative example of the side effects of anticancer drugs with well-described causal relationship is anthracycline-induced cardiotoxicity [5,6]. Anthracyclines cause damage to myocardial cells via free radical generation [7]. As for the types of anthracycline-induced cardiotoxicity, acute cardiotoxicity occurs in <1% of the patients immediately after drug infusion, the early-onset type occurs in 1.6%–2.1% of patients during treatment or within the first year after treatment, and the late-onset type occurs in 1.6%–5% of patients at least 1 year after completion of chemotherapy [8]. Trastuzumab, which contributes to a remarkable increase in the survival rate of patients with human epidermal growth factor receptor-2 (HER2)-positive (+) early BC, is also among the widely known cardiotoxic agents [9,10]. A high incidence of severe congestive heart failure (CHF) was reported in the Herceptin Adjuvant (HERA) trial, one of the largest clinical studies on trastuzumab [11]. Severe cardiotoxicity following treatment with trastuzumab was reported in 3% of the patients in a meta-analysis of 29,000 women with BC [12]. Anthracyclines and trastuzumab have different cardiotoxic mechanisms; therefore, the concurrent use of the two agents has been reported to be associated with an increased risk of cardiotoxicity [13]. In a phase 3 trial on the comparative evaluation of the efficacy and safety of the standard chemotherapy alone (doxorubicin and cyclophosphamide) and standard chemotherapy plus trastuzumab in patients with metastatic BC, 27% of the patients in the combination therapy arm experienced cardiotoxicity, whereas only 8% of the patients in the monotherapy arm experienced cardiotoxicity [14]. Other studies have reported an increased risk of cardiac toxicity in some patients with the combined use of anthracyclines and trastuzumab [14,15]. Thus, the concomitant administration of the two agents is contraindicated and a sequential strategy of administration is considered in a limited number of cases [16]. In addition to anthracycline- or trastuzumab-induced cardiotoxicity, radiotherapy, diabetes mellitus, and hypertension are the major risk factors for cardiovascular disease (CVD) in patients with BC [17–19].

To monitor and prevent cardiotoxicity, which has a serious impact on mortality rate and the quality of life of BC survivors, various recommendations have been proposed in clinical practice guidelines [2,20–22]. A recommendation is to limit the cumulative total lifetime dose of anthracyclines, which induces dose-dependent cardiotoxicity [23]. For early detection, patients should undergo routine electrocardiogram and left ventricular ejection fraction (LVEF) monitoring with echocardiography [24]. Furthermore, the underlying risk factors in individual patients and preventive treatments for high-risk populations should be considered [25]. Although various strategies are used as recommended by these clinical guidelines, further studies are needed to verify and confirm their effectiveness in preventing cardiotoxicity in real-world practice [26]. In addition, extensive research and better understanding are required for cases with little clinical manifestation of the symptoms or for the risk of late-onset, long-term cardiotoxicity [27–29]. Therefore, there is a pressing need for the accumulation of more clinical evidence that would enable the identification of patients at a higher risk of CVD and for subsequently establishing a treatment strategy to reduce the risk of developing CVD.

For CVD, which is the leading cause of mortality among patients with BC other than the cancer itself, a number of previous studies investigated the incidence and clinical predictors (including exposure to a high cumulative doxorubicin dose, trastuzumab use, and chest radiotherapy) of anticancer agent-induced cardiotoxicity in treatment of BC [21,30]. However, according to a systematic review on the risk of death from CVD following BC, there were limitations in the generalizability of findings because published studies were heterogeneous in terms of their study design, population, study period, age of patients included in the analysis, and cancer stage of the patients included in the analysis. The number of included studies was also small [31]. Therefore, in this study, a claims database, which includes a nationwide cohort of patients with BC in Korea, was used for a detailed analysis. For the study population of all patients with BC in Korea, long-term follow-up monitoring was conducted for outcomes of CVD events and all-cause mortality following anthracycline therapy, and risk factors influencing the occurrence of the outcomes were evaluated.

## Methods

### Data source

This nationwide, retrospective cohort study used healthcare claims data retrieved from the National Health Insurance Service (NHIS) of Korea. The database covers approximately 50 million people (99% of the South Korean population) from January 1, 2002, to December 31, 2020. The recorded information includes details on sex, age, inpatient and outpatient medical services, diagnostic codes, drug prescriptions, and healthcare providers. This database is nationally representative and includes the dates of death of the population from Korean Statistics. Cause-of-death information was not available in the claims extract; thus, mortality analyses were limited to all-cause deaths. The related materials and metadata are publicly available on the National Health Insurance Data Sharing Service homepage (http://nhiss.nhis.or.kr (accessed on October 6, 2023)). The Institutional Review Board of Gachon University granted a formal waiver for consent (no.: 1044396–202101-HR-001–01).

### Study cohort

**Fig 1:**
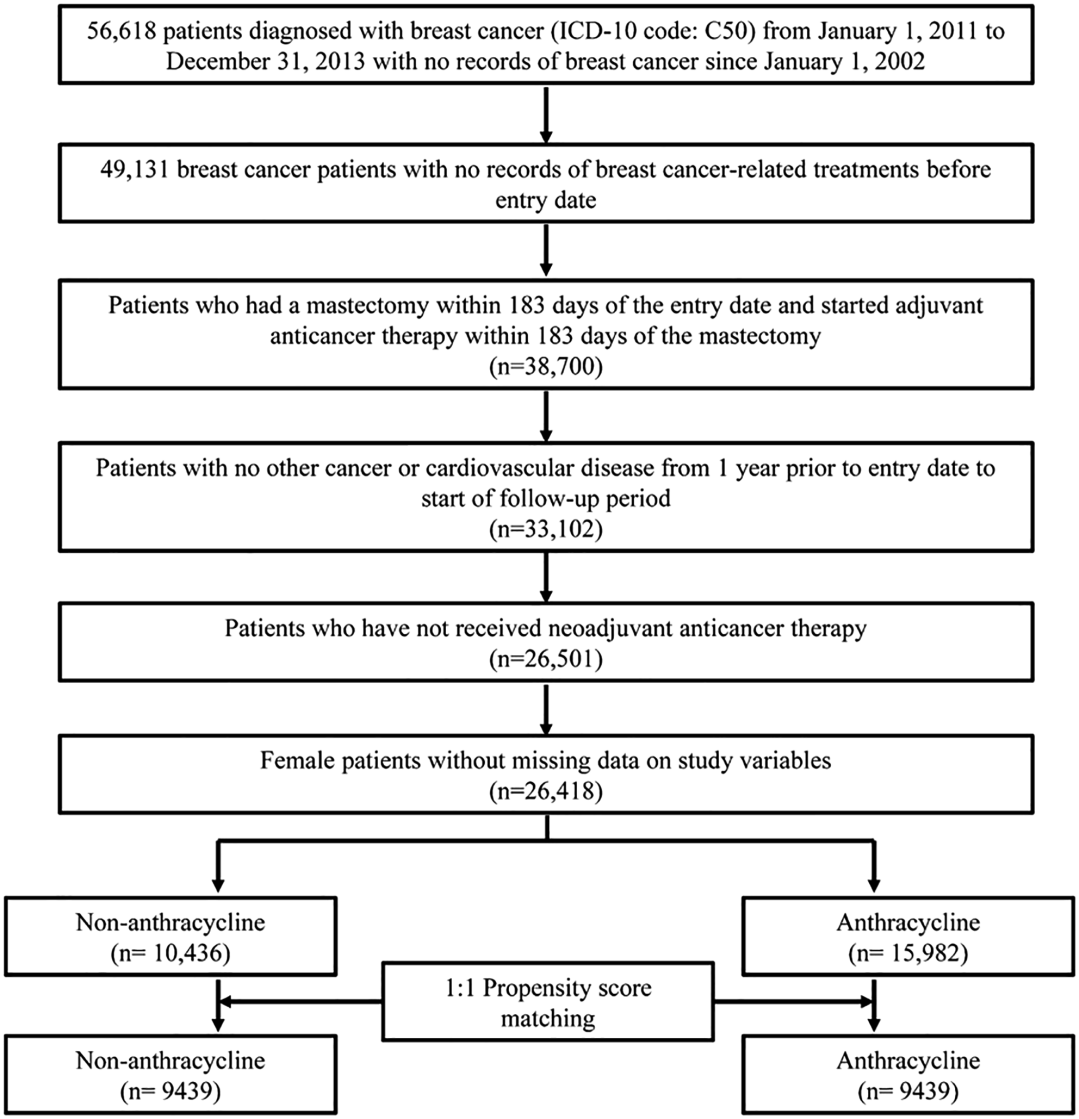
Study population flowchart. ICD-10: International classification of diseases 10th revision.

**Fig 2:**
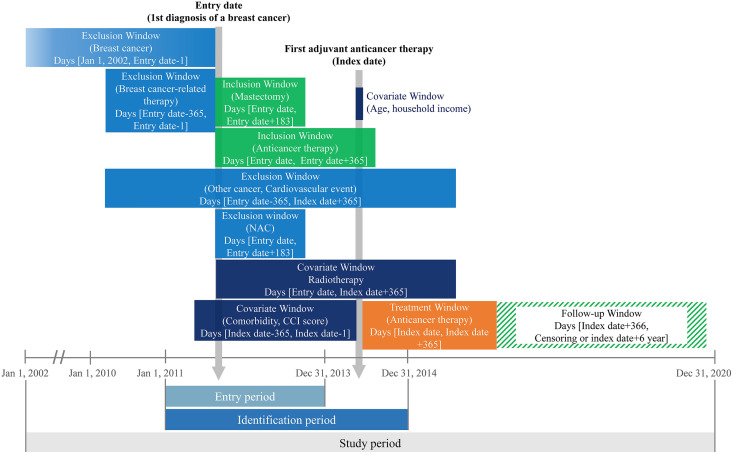
Study design. NAC: neoadjuvant cancer therapy; CCI: Charlson comorbidity index.

The National Health Insurance (NHI) claims data from January 1, 2002, were used, and patients who were first diagnosed with BC (International Classification of Diseases 10th Revision [ICD-10] code: C50) between January 1, 2011, and December 31, 2013, were included in the study cohort; the date of the first diagnosis of BC was defined as the entry date. To control for the BC stage and disease-related characteristics of the patients included in the study cohort, those who underwent mastectomy within 6 months after the first diagnosis of BC and received chemotherapy within 183 days after mastectomy were included. The exclusion criteria were as follows: [1] patients who underwent treatment that may be considered BC treatment (anticancer therapy [chemotherapy], mastectomy, radiotherapy) during the year preceding the entry date; [2] patients who received neoadjuvant chemotherapy; [3] patients with a history of cancer other than BC or CVD from 1 year preceding the entry date to the start of follow-up; and [4] patients with missing data in the study variables and male patients (Figs 1, 2).

## Exposure

In this study, adjuvant anthracycline therapy as anticancer therapy for BC was defined as exposure. The study cohort design consisted of an anthracycline group for patients who received at least one prescription of anthracycline therapy within 1 year from the date of the first adjuvant anticancer therapy (defined as the index date) and a non-anthracycline group for patients who did not receive anthracycline therapy. The treatment window was defined as 1 year considering the treatment period of anthracyclines and other agents used as anticancer therapy for BC [32] with the aim of evaluating the risk of early onset and late-onset chronic progressive cardiotoxicity, excluding acute cardiotoxicity cases [8]. Anthracyclines included chemotherapeutic agents with doxorubicin or epirubicin that were listed for NHI reimbursement during the study period. Anthracycline exposure was defined as any prescription of doxorubicin or epirubicin within 365 days of the index date; cumulative dose (e.g., mg/m²) and dose intensity were not available in the claims data and were therefore not incorporated. Owing to the increased risk of heart failure (HF) in cases of concomitant administration with anthracyclines, concurrent administration of trastuzumab was contraindicated. However, since trastuzumab, whose sequential administration following the adjuvant anthracycline therapy may be considered in clinical practice, is a major influencing factor for the evaluation of outcomes in this study, subgroup analysis was performed by grouping the patients according to adjuvant trastuzumab status.

### Study outcomes and covariates

CVD events were defined as cases of NHI claims with diagnostic codes of coronary artery disease (CAD) and cardiac arrest, CHF, HF and cardiomyopathy, or stroke (ST) within the 5th-listed secondary diagnosis during the follow-up period. The diagnostic codes defined as the outcomes of this study were based on the ICD-10, and the details are provided in S1 Table. The primary outcomes of interest were the incidence of the composite cardiovascular events and all-cause death during the study period, and the evaluation of risk factors affecting the incidence of these events. The primary composite cardiovascular outcome was defined as time to the first occurrence of any of the following subtypes: coronary artery disease and cardiac arrest, congestive heart failure, heart failure and cardiomyopathy, or stroke; follow-up ended at the earliest of the first CVD event, death, or end of follow-up. For secondary outcomes, we conducted separate time-to-event analyses for each subtype; occurrences of other CVD subtypes did not censor follow-up for the subtype under analysis, and recurrent events within the same subtype were not counted.

From the nationwide cohort database in this study, measurable sociodemographic characteristics (age and household income quintile), Charlson comorbidity index (CCI) scores, and comorbidities related to the incidence of outcomes were included as covariates [33–35]. Information on the age and household income quintiles of patients with BC was collected from NHI claims issued on the index date, and comorbidities and CCI scores were evaluated from claims issued during the year preceding the index date. The status of adjuvant trastuzumab, which is associated with cardiotoxicity, within 1 year from the index date [10] and the history and frequency of radiotherapy from the entry date (date of the first diagnosis of BC) to 1 year from the index date were included as covariates in this study [17]. In addition, the frequency of adjuvant anticancer therapy administered within 1 month of the index date for each patient group was documented and is presented in the supplementary.

### Statistical analysis

Differences in baseline characteristics between the anthracycline and non-anthracycline groups were compared using the Chi-square test. The incidence rate (per 100 person-years) was derived by measuring the incidence of the defined outcomes for each patient group and the time to onset for each incidence. The incidence rate ratio (IRR) and 95% confidence interval (CI) were also calculated. Follow-up was defined as monitoring up to the earliest time point of the occurrence of a CVD event, death, or the end of the 5-year follow-up period. Accordingly, follow-up began 365 days after the index date (the first adjuvant anticancer therapy) and continued until the earliest occurrence of a composite cardiovascular event, death, or 5 years. Participants without an event were censored at 5 years or at the end of available data, whichever came first. For secondary outcomes, we conducted separate cause-specific time-to-first-event analyses for each subtype; events of other CVD subtypes did not censor follow-up for the subtype under analysis, allowing a single patient to contribute at most one first event to multiple different subtypes. For analysis of risk factors for CVD and mortality of the patients with BC, the Cox proportional hazards model was used to calculate hazard ratios (HR) and 95% CIs. Proportional hazards were assessed by visual inspection of Kaplan–Meier curves for the exposure groups; no major departures (e.g., curve crossing) were observed. In the Cox model, exposure to adjuvant anthracycline therapy, CCI score, household income quintile, presence of adjuvant trastuzumab, comorbidities, and frequency of radiotherapy were included as covariates. Patients were grouped into subgroups of those who underwent adjuvant trastuzumab therapy within 1 year from the index date and those who did not, and a subgroup analysis was performed to evaluate the difference in the trend of CVD event incidence. A 1:1 greedy propensity score matching was performed for age group (exact variable), household income quantile, CCI score, and number of radiotherapies. Differences in characteristics between the patient groups before and after propensity score matching were analyzed using Chi-square tests. Statistical significance was defined as two-sided p < 0.05. All the statistical analyses were performed using SAS version 9.4 (SAS Institute, Cary, NC, USA).

## Results

### Baseline characteristics

From a cohort of 56,618 patients who were first diagnosed with BC between 2011 and 2013, 9,439 patients were included in both the anthracycline and non-anthracycline groups after a final screening based on the inclusion and exclusion criteria. (Table 1) The age group included as an exact matching variable in the propensity score matching showed an identical distribution between the two groups, and the distribution of other variables showed similarity between the two groups after matching. However, statistically significant between-group differences in these variables were still observed. In terms of comorbidities, higher proportions of other CVDs and sleep disorders occurred in the anthracycline group (4.29% non-anthracycline vs. 5.37% anthracycline; 12.71% non-anthracycline vs. 14.30% anthracycline, respectively), whereas osteoporosis showed higher prevalence in the non-anthracycline group (30.59% non-anthracycline vs. 25.46% anthracycline). Most other comorbidities showed similar trends between the two groups (Table 2; extended variables in S2 Table). In the case of the non-anthracycline group, as the adjuvant anticancer therapy for the first year, selective estrogen receptor modulators (SERM) (64.11% vs. 44.76%), aromatase inhibitors (AI) (30.58% vs. 25.01%), and methotrexate (20.18% vs. 0.19%) were used by higher percentages whereas taxanes (1.33% vs. 38.73%) were used by a small percentage of the patients (S3 Table).

**Table 1:** Patient selection cascade.

Exclusion reason	No. of excluded patients
Prior breast cancer–related treatments before entry date	7,487
Did not meet timing: mastectomy within 183 days of the entry date and adjuvant therapy within 183 days of the mastectomy	10,431
Other cancer or cardiovascular disease within 1 year before entry to start of follow-up	5,598
Received neoadjuvant anticancer therapy	6,601
Male sex or missing data in study variables	83
Unmatched in 1:1 propensity score matching	7,540

**Table 2:** Baseline characteristics (condensed).

Variables	Before matching					After matching				
	Non-anthracycline (n = 10,436)		Anthracycline (n = 15,982)		P-value	Non-anthracycline (n = 9439)		Anthracycline (n = 9439)		P-value
	N	%	N	%		N	%	N	%	
**Age groups**					<.0001					1
<45	2526	24.2	4931	30.85		2495	26.43	2495	26.43	
45–54	4136	39.63	6881	43.05		4109	43.53	4109	43.53	
55–64	2064	19.78	3234	20.24		1983	21.01	1983	21.01	
≥65	1710	16.39	936	5.86		852	9.03	852	9.03	
**CCI score**					<.0001					0.0025
2	3902	37.39	6308	39.47		3705	39.25	3480	36.87	
3–4	4687	44.91	6210	38.86		4209	44.59	4335	45.93	
≥5	1847	17.7	3464	21.67		1525	16.16	1624	17.21	
Adjuvant trastuzumab*	386	3.7	3429	21.46	<.0001	335	3.55	2036	21.57	<.0001
**Comorbidity**										
Diabetes mellitus	1282	12.28	1459	9.13	<.0001	977	10.35	911	9.65	0.1094
Hyperlipidemia	3078	29.49	4078	25.52	<.0001	2591	27.45	2529	26.79	0.3101
Hypertension	2377	22.78	2890	18.08	<.0001	1809	19.17	1873	19.84	0.2398
Other cardiovascular diseases	486	4.66	818	5.12	0.0906	405	4.29	507	5.37	0.0005
Renal failure	68	0.65	74	0.46	0.0404	56	0.59	41	0.43	0.1268
Cerebrovascular disease	204	1.95	203	1.27	<.0001	158	1.67	122	1.29	0.0302
Radiotherapy*					<.0001					<.0001
Yes	7824	74.97	12497	78.19		7258	76.89	7564	80.14	

CCI: Charlson comorbidity index; COPD: chronic obstructive pulmonary disease; Full list of baseline variables is provided in S2 Table.

### Analysis of incidence

The incidence rate of the composite cardiovascular events was 1.864 per 100 person-years for the anthracycline group and 1.958 per 100 person-years for the non-anthracycline group, and the IRR was 0.952 (95% CI 0.866–1.046), confirming no significant between-group differences. As a result of analyzing the incidence rates of the four types of CVD events in the anthracycline group, CHF (IRR 1.185 [95% CI 1.013–1.386]) and HF and cardiomyopathy (IRR 1.197 [95% CI 1.008–1.421]) showed high incidence rates, and CAD and cardiac arrest (IRR 0.900 [95% CI 0.794–1.021]) and ST (IRR 0.842 [95% CI 0.686–1.033]) showed relatively low incidence rates, but no statistically significant differences were confirmed. In contrast, the incidence of all-cause mortality was 1.434 per 100 person-years in the anthracycline group and 0.684 per 100 person-years in the non-anthracycline group, indicating a higher incidence of all-cause mortality in the anthracycline group (IRR 2.155 [95% CI 1.892–2.455]) (Table 3).

**Table 3:** Incidence rate for study outcomes.

Outcome	Group	No. of events	Person-year	Incidence rate per 100 person-years	Incidence rate ratio (95% CI)	P-value
**Composite cardiovascular event**	Anthracycline	839	45,010	1.864	0.952 (0.866–1.046)	0.3067
	Non-anthracycline	893	45,610	1.958		
Coronary artery disease and cardiac arrest	Anthracycline	474	45,970	1.031	0.906 (0.800–1.026)	0.1182
	Non-anthracycline	529	46,477	1.138		
Congestive heart failure	Anthracycline	331	46,235	0.716	1.110 (0.950–1.297)	0.1894
	Non-anthracycline	304	47,130	0.645		
Heart failure and cardiomyopathy	Anthracycline	277	46,379	0.597	1.125 (0.948–1.334)	0.1773
	Non-anthracycline	251	47,269	0.531		
Stroke	Anthracycline	171	46,687	0.366	0.857 (0.699–1.051)	0.1376
	Non-anthracycline	202	47,265	0.427		
**All-cause mortality**	Anthracycline	707	47,057	1.502	2.155 (1.892–2.455)	<.0001
	Non-anthracycline	333	47,763	0.697		

### Analysis of hazards

**Fig 3:**
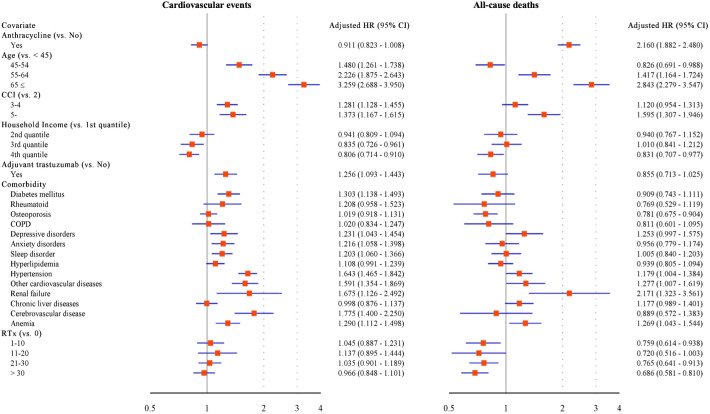
Risk factors for cardiovascular events and mortality in patients with breast cancer. HR, hazard ratio; CCI, Charlson comorbidity index; COPD: Chronic obstructive pulmonary disease; RTx, radiotherapy.

Similar to the results from the analysis of incidence rates, the results of Cox proportional hazards analysis showed that adjuvant anthracyclines administered in the first year did not have a significant impact on the risk of the composite cardiovascular events (adjusted HR 0.911 [95% CI 0.823–1.008]); however, the adjuvant anthracycline therapy increased the risk of all-cause mortality (adjusted HR 2.160 [95% CI 1.882–2.480]). Older age, high CCI scores, comorbidities of other CVDs, anemia, renal failure, and hypertension were identified as factors contributing to an increased risk of the composite cardiovascular events and all-cause mortality. Patients in the high household income quintile showed a lower risk of both outcomes. When the patients underwent adjuvant trastuzumab therapy in the first year, the risk of the composite cardiovascular events was high (aHR, 1.256 [95% CI, 1.093–1.443]) (Fig 3). Adjuvant trastuzumab increased the risk of CHF (aHR 1.715 [95% CI 1.395–2.108]) and HF and cardiomyopathy (aHR 1.871 [95% CI 1.498–2.335]), but the agent had no significant impact on the risk of CAD and cardiac arrest or ST (Table 4).

**Table 4:** Multivariable Cox regression analysis for cardiovascular events.

	Coronary artery disease and cardiac arrest	Congestive heart failure	Heart failure and cardiomyopathy	Stroke
	aHR (95% CI)	aHR (95% CI)	aHR (95% CI)	aHR (95% CI)
**Anthracycline (vs. No)**				
Yes	0.940 (0.823–1.074)	0.977 (0.825–1.157)	0.971 (0.806–1.169)	0.902 (0.725–1.122)
**Age (vs. < 45)**				
45–54	1.646 (1.329–2.040)	1.107 (0.854–1.435)	1.184 (0.891–1.575)	1.689 (1.151–2.479)
55–64	2.395 (1.905–3.011)	1.762 (1.339–2.319)	1.899 (1.404–2.568)	2.829 (1.895–4.221)
≥65	3.238 (2.509–4.179)	2.720 (2.011–3.681)	2.901 (2.077–4.050)	5.411 (3.527–8.301)
**CCI score (vs. 2)**				
3–4	1.306 (1.107–1.541)	1.274 (1.036–1.566)	1.199 (0.960–1.498)	1.103 (0.841–1.446)
≥5	1.456 (1.183–1.793)	1.429 (1.101–1.854)	1.341 (1.010–1.781)	0.958 (0.670–1.371)
**Household income quantile (vs. 1st quantile)**				
2nd quantile	0.891 (0.728–1.091)	1.124 (0.864–1.463)	1.129 (0.845–1.508)	1.020 (0.745–1.397)
3rd quantile	0.819 (0.679–0.988)	1.038 (0.813–1.325)	1.047 (0.800–1.369)	0.802 (0.597–1.079)
4th quantile	0.808 (0.680–0.959)	1.037 (0.827–1.301)	1.044 (0.814–1.340)	0.649 (0.490–0.861)
**Adjuvant trastuzumab (vs. No)**				
Yes	1.038 (0.858–1.255)	1.784 (1.456–2.186)	1.884 (1.511–2.349)	1.051 (0.772–1.432)
**Comorbidity**				
Diabetes mellitus	1.278 (1.074–1.522)	1.052 (0.842–1.314)	0.947 (0.735–1.219)	1.441 (1.087–1.909)
Rheumatoid	1.138 (0.839–1.543)	1.224 (0.842–1.780)	1.248 (0.828–1.882)	1.605 (1.021–2.523)
Osteoporosis	1.137 (0.995–1.299)	1.020 (0.861–1.209)	1.067 (0.886–1.284)	0.993 (0.796–1.239)
COPD	0.929 (0.707–1.222)	0.972 (0.702–1.346)	0.906 (0.626–1.314)	0.703 (0.436–1.134)
Depressive disorders	1.350 (1.090–1.671)	1.372 (1.050–1.792)	1.280 (0.952–1.721)	1.086 (0.758–1.555)
Anxiety disorders	1.335 (1.118–1.594)	0.992 (0.780–1.261)	1.018 (0.784–1.323)	1.071 (0.791–1.450)
Sleep disorder	1.029 (0.867–1.223)	1.112 (0.899–1.374)	1.245 (0.992–1.562)	1.586 (1.229–2.045)
Hyperlipidemia	1.231 (1.065–1.424)	1.169 (0.974–1.402)	1.110 (0.909–1.357)	1.030 (0.810–1.309)
Hypertension	1.524 (1.312–1.769)	2.183 (1.810–2.632)	2.023 (1.647–2.483)	1.654 (1.295–2.113)
Other cardiovascular diseases	1.387 (1.114–1.726)	1.963 (1.547–2.490)	2.065 (1.594–2.674)	0.839 (0.555–1.269)
Renal failure	1.929 (1.144–3.252)	3.391 (2.076–5.538)	3.306 (1.897–5.761)	2.628 (1.211–5.705)
Chronic liver diseases	1.064 (0.902–1.255)	0.823 (0.660–1.025)	0.845 (0.663–1.078)	1.033 (0.782–1.364)
Cerebrovascular disease	1.092 (0.765–1.558)	1.174 (0.772–1.785)	1.385 (0.893–2.150)	3.890 (2.691–5.623)
Anemia	1.155 (0.947–1.409)	1.341 (1.055–1.704)	1.389 (1.069–1.804)	1.207 (0.866–1.683)
**Radiotherapy (vs. 0)**				
1–10	1.063 (0.855–1.322)	1.007 (0.766–1.324)	0.962 (0.708–1.308)	1.051 (0.747–1.479)
11–20	1.254 (0.935–1.682)	1.255 (0.872–1.808)	1.419 (0.965–2.086)	0.682 (0.389–1.196)
21–30	1.023 (0.852–1.229)	1.002 (0.800–1.255)	1.041 (0.813–1.332)	1.040 (0.784–1.381)
≥31	1.046 (0.884–1.239)	0.970 (0.787–1.197)	0.997 (0.792–1.256)	0.845 (0.642–1.112)

aHR: adjusted hazard ratio; CCI: Charlson comorbidity index; CI: confidence interval; COPD: chronic obstructive pulmonary disease.

As a result of subgroup analysis by grouping the patients into subgroups according to the use of adjuvant trastuzumab as the first year adjuvant anticancer therapy, among patients without adjuvant trastuzumab (n = 22,603 eligible), the propensity score–matched cohort comprised 9,093 patients per group (total 18,186), in whom mortality was significantly higher in the anthracycline group (aHR 2.335 [95% CI 2.027–2.689]). Advanced age and renal failure increased the risk of most outcomes, showing a similar trend as in the main analysis; however, there was a difference in that the risk of outcomes increased with higher income quintile of the patients (S4 Table). Among patients who received adjuvant trastuzumab (n = 3,815 eligible), the propensity score–matched cohort included 386 patients per group (total 772); within this matched set, adjuvant anthracyclines were not a significant predictor for any outcome. Similar to the results of the other analyses described above, advanced patient age increased the risk of most outcomes (S5 Table).

## Discussion

In this study, the outcomes of the composite cardiovascular events and all-cause mortality were compared between patients administered and those not administered anthracycline treatment among those who underwent adjuvant anticancer therapy within 1 year after the first diagnosis of BC. In addition, the risk factors affecting the incidence of the outcomes were evaluated. The incidence rates of CHF, HF and cardiomyopathy, and all-cause mortality were higher in the anthracycline group. Advanced age, high CCI score, anemia, and renal failure were common major risk factors for the composite cardiovascular events and mortality. Adjuvant trastuzumab therapy was a risk factor for CHF and HF and cardiomyopathy; however, when subgroup analysis was performed considering the presence of adjuvant trastuzumab, adjuvant anthracyclines were not a significant risk factor for all outcomes. Given the large sample size, small differences may achieve statistical significance; therefore, we emphasized effect sizes with 95% CIs and clinical relevance over p-values and reported absolute incidence rates to aid interpretation.

The prognosis of BC with both hormone receptors (estrogen receptor [36] or progesterone receptor [PR]) positive (ER + /PR+) is better than that with only one hormone receptor positive or both hormone receptors negative, as the tumor phenotypes in the latter cases are more aggressive and have a more unfavorable prognosis [37]. In the case of ER + /PR + BC, endocrine therapy such as AI or SERM is considered the first-line therapy. Thus, there are more patients with hormone receptor-positive BC in the non-anthracycline group, which resulted in a lower mortality rate. In this study, the frequency of the concomitant administration of different anticancer agents between the two groups showed that a high proportion of patients in the non-anthracycline group received AI and SERM therapies (S3 Table). Many of the patients in the anthracycline group also underwent endocrine therapy, and these are presumed to be patients with metastatic BC who had metastasis after endocrine therapy or whose treatment failed due to resistance to the endocrine therapy [38], which may have contributed to increasing the risk of mortality in the anthracycline group [37]. Beyond differences in endocrine therapy, residual confounding by indication remains a plausible explanation: anthracyclines are often prescribed for biologically higher‐risk disease, and unmeasured tumor characteristics (e.g., stage, grade, burden) not captured in claims data may have adversely influenced prognosis despite propensity score matching and adjustment [39]. Additionally, subclinical or non-coded cardiotoxicity—manifesting as asymptomatic declines in left-ventricular ejection fraction—may prompt treatment modification without being captured by ICD-coded cardiovascular endpoints, thereby indirectly contributing to poorer cancer outcomes [40].

In the present study, based on the analysis of real-world data, the unadjusted incidence rates of CHF and HF and cardiomyopathy in patients with BC treated with adjuvant anthracyclines were found to be high; however, Cox proportional hazard analysis adjusted for several covariates showed that the effect of adjuvant anthracyclines on the outcomes was not significant. Anthracycline-induced cardiotoxicity, a side effect of chemotherapy agents, was reported several decades ago, and methods of active monitoring and prevention of chemotherapy-induced cardiotoxicity have been under development [2,5,6,24]. Thus, recent studies on the treatment of patients with BC report that the side effects do not progress to severe cardiotoxicity. In the patient cohort of the present study, CVDs other than cardiotoxicity (CAD and cardiac arrest, and ST) did not show a specific trend. Unlike adjuvant anthracyclines, adjuvant trastuzumab therapy has a significant impact on the incidence of CHF and HF and cardiomyopathy. Trastuzumab-induced cardiotoxicity is also a commonly reported side effect [11], and the findings of this study also showed consistency in this aspect; however, the results of the subgroup analysis based on subgroups depending on the adjuvant trastuzumab status showed no statistical significance in terms of the risk of cardiotoxicity (S4 and S5 Tables). Subgroup analysis can be considered as an analysis based on the types of receptor expression between groups, consisting of the HER-2 overexpression group, for whom adjuvant trastuzumab is recommended, and the group of the other patients without HER-2 overexpression [41]. In each subgroup, adjuvant anthracycline therapy was not a significant risk factor for incidence rates of CHF or HF and cardiomyopathy. Although the sample size was small, the administration of anthracycline and trastuzumab (concomitant or sequential) in the first year of adjuvant therapy was not significantly associated with the risk of CHF and HF and cardiomyopathy.

With increased overall survival after treatment, CVD is now considered one of the leading causes of death in patients with BC. A previous study reported that BC-related and other-cause deaths increased with age and in patients with CVD [42], which is consistent with the findings of this study. Although few studies have been published on renal disease in patients with BC, the findings of this study show that renal disease, one of the major risk factors for CHF, is associated with an increased risk of cardiovascular events and mortality [43]. This study also showed that adjuvant radiotherapy was associated with a reduced risk of all-cause mortality, which is similar to that reported in previous studies [44,45]. On the contrary, a previous study reported that adjuvant radiotherapy induced cardiovascular complications and increased mortality from cardiovascular causes [46], and the difference between the findings of this study and those of the previous study may be because of the fact that the duration of follow-up in this study was not sufficiently long for evaluation of radiotherapy-induced CVDs. In addition, although not included in the analysis, active research is underway on cardioprotective therapies (e.g., beta-blockers, statins, or dexrazoxane) that may reduce the risk of CVD and death in patients with BC [47].

With advancements in diagnostic techniques and the development of personalized treatments for BC, the treatment patterns of individual real-world patients with BC are becoming highly diverse [48]. Thus, even if studies use the same outcomes for assessment, patient characteristics are heterogeneous in each prior study, resulting in heterogeneous results among these studies and posing difficulties in comparisons between studies. The significance of this study lies in the comparison of incidences of the CVD events and all-cause mortality following adjuvant anthracycline therapy for patients with BC who underwent mastectomy during the early period from diagnosis without neoadjuvant anticancer therapy, as well as risk factors for the outcomes, which contributed to establishing clinical evidence that can be used as basic reference data for BC treatment in real-world clinical settings. In the future, using data sources across multiple countries and those containing a wide range of patient information such as cancer stage and laboratory test results, follow-up studies are warranted to obtain clinical evidence considering the various characteristics of real-world patients with BC.

This study analyzed the incidence of outcomes in patients with BC in Korea using NHI claims data. By ensuring a follow-up period of up to 5 years per patient, which allowed the monitoring of long-term effects, late-onset cardiotoxicity in patients with BC was evaluated. The incidence of the composite cardiovascular events, rather than a decrease in LVEF, an indicator of early cardiotoxicity, was used as the outcome, which enabled the evaluation of the incidence of severe cardiotoxicity. Furthermore, the incidences of CAD and cardiac arrest, and ST, which are CVDs other than cardiotoxicity, were presented, which allowed the detection of any bias from the characteristics of the patient cohort.

This study also has several limitations. First, data such as BC stage, laboratory data, and lifestyle factors could not be collected for each patient because these characteristics cannot be collected from the NHI claims data. To mitigate selection bias, we restricted the cohort to patients who underwent mastectomy within 6 months of the first BC diagnosis without neoadjuvant therapy, and who initiated adjuvant anticancer therapy within 6 months after surgery. Baseline characteristics were balanced using PSM. Some residual imbalance remained; however, in large samples, small absolute differences may remain statistically significant. We further adjusted for covariates using multivariable Cox models in the matched cohort, but residual and unmeasured confounding cannot be ruled out. Furthermore, formal proportional-hazards diagnostics were not conducted (e.g., Schoenfeld residuals), and although Kaplan–Meier curves did not demonstrate gross crossing, visual inspection could not exclude modest non-PH. Consequently, reported HRs should be interpreted as average effects over follow-up [49]. Subsequent analyses will incorporate formal PH tests and, where appropriate, alternatives such as time-varying Cox specifications or restricted mean survival time. Second, the absence of tumor stage and molecular subtype—key determinants of treatment and prognosis—may introduce confounding by indication and limit risk stratification and generalizability. In lieu of receptor data, we used adjuvant trastuzumab prescriptions as a pragmatic proxy for HER2 status; although its positive predictive value is likely high, this indirect measure may miss some HER2-positive patients not treated with trastuzumab (e.g., older or comorbid individuals who did not receive trastuzumab), leading to misclassification [50]. Subgroup findings should therefore be interpreted with caution, and linkage to tumor registries or EHRs to confirm biomarkers is warranted. Third, anthracycline exposure was classified as a binary status (ever/never) within the 1-year treatment window; cumulative dose (e.g., mg/m²) and dose intensity were not captured. Given the dose–response relationship for anthracycline cardiotoxicity, this may have attenuated dose-dependent associations, and estimates should be interpreted with caution. In addition, secondary subtype analyses were modeled separately without a competing-risks or multi-state framework; other CVD subtypes were not treated as competing events, and multiple subtype-specific tests raise concerns about multiplicity. Lastly, due to the retrospective design and prespecified treatment window, anticancer therapies initiated or continued outside the window could not be fully accounted for. This prespecified 1-year window was chosen to harmonize exposure ascertainment across regimens and to align with the intensive adjuvant period during which anthracyclines are typically administered, thereby enhancing comparability and minimizing time-related ambiguities in claims data. Accordingly, our estimates primarily reflect risks in the early and early-chronic post-therapy periods; future studies using time-varying exposure frameworks can build on these findings to evaluate longer horizons.

## Conclusion

Analysis of real-world data from a nationwide cohort of patients with BC in Korea showed that the incidence rates of CHF, HF and cardiomyopathy, and all-cause mortality were higher among patients who received adjuvant anthracycline therapy as the initial anticancer therapy. However, the results of the survival analysis adjusted for related covariates showed that adjuvant anthracyclines were not a significant risk factor for the composite cardiovascular events specified in this study. Older patients and those with high CCI scores and comorbidities of renal failure may have higher risks of cardiotoxicity and mortality, indicating that more thorough and well-planned monitoring is required for patients with these characteristics.

## Supporting information

S1 Table Study information (outcome definitions and ICD-10 codes). (DOCX)

S2 Table Baseline characteristics (extended). (DOCX)

S3 Table Frequency of adjuvant anticancer therapy administered within 1 month of the index date. (DOCX)

S4 Table Multivariable Cox regression analysis for cardiovascular events in patients not treated with trastuzumab. (DOCX)

S5 Table Multivariable Cox regression analysis for cardiovascular events in patients treated with trastuzumab. (DOCX)

## Abbreviations

IRR: incidence rate ratios
HR: hazard ratios
CI: confidence intervals
BC: breast cancer
HER2: human epidermal growth factor receptor-2
CHF: congestive heart failure
HERA: Herceptin Adjuvant
CVD: cardiovascular disease
LVEF: left ventricular ejection fraction
NHIS: National Health Insurance Service
NHI: National Health Insurance
NAC: neoadjuvant chemotherapy
ICD-10: International Classification of Diseases 10th Revision
CAD: coronary artery disease
HF: heart failure
ST: stroke
CCI: Charlson comorbidity index
SERM: selective estrogen receptor modulators
AI: aromatase inhibitors
ER: estrogen receptor
PR: progesterone receptor
COPD: chronic obstructive pulmonary disease
RTx: radiotherapy
