# Supplementary material for: Risk of cardiovascular disease and death in patients with breast cancer receiving anthracycline-based therapy: A retrospective cohort study
Source: PLoS One. 2025 Dec 4;20(12):e0335083. doi: 10.1371/journal.pone.0335083 (PMC12677787; doi:10.1371/journal.pone.0335083)
Supplement: S2 Table — (DOCX) [file pone.0335083.s002.docx]

**Supplementary Table S2. Baseline characteristics (extended)**

| **Variables** | **Before matching** | | | | | **After matching** | | | | |
| --- | --- | --- | --- | --- | --- | --- | --- | --- | --- | --- |
|  | **Non-anthracycline (n = 10,436)** | | **Anthracycline (n = 15,982)** | | **P-value** | **Non-anthracycline (n = 9439)** | | **Anthracycline (n = 9439)** | | **P-value** |
|  | **N** | **%** | **N** | **%** |  | **N** | **%** | **N** | **%** |  |
| **Age groups** |  |  |  |  | <.0001 |  |  |  |  | 1 |
| <45 | 2526 | 24.2 | 4931 | 30.85 |  | 2495 | 26.43 | 2495 | 26.43 |  |
| 45–54 | 4136 | 39.63 | 6881 | 43.05 |  | 4109 | 43.53 | 4109 | 43.53 |  |
| 55–64 | 2064 | 19.78 | 3234 | 20.24 |  | 1983 | 21.01 | 1983 | 21.01 |  |
| ≥65 | 1710 | 16.39 | 936 | 5.86 |  | 852 | 9.03 | 852 | 9.03 |  |
| **CCI score** |  |  |  |  | <.0001 |  |  |  |  | 0.0025 |
| 2 | 3902 | 37.39 | 6308 | 39.47 |  | 3705 | 39.25 | 3480 | 36.87 |  |
| 3–4 | 4687 | 44.91 | 6210 | 38.86 |  | 4209 | 44.59 | 4335 | 45.93 |  |
| ≥5 | 1847 | 17.7 | 3464 | 21.67 |  | 1525 | 16.16 | 1624 | 17.21 |  |
| **Household income quantile** |  |  |  |  | <.0001 |  |  |  |  | <.0001 |
| 1st quantile | 2393 | 22.93 | 3801 | 23.78 |  | 2173 | 23.02 | 2173 | 23.02 |  |
| 2nd quantile | 1704 | 16.33 | 3048 | 19.07 |  | 1586 | 16.8 | 1487 | 15.75 |  |
| 3rd quantile | 2252 | 21.58 | 3726 | 23.31 |  | 2080 | 22.04 | 1900 | 20.13 |  |
| 4th quantile | 4087 | 39.16 | 5407 | 33.83 |  | 3600 | 38.14 | 3879 | 41.1 |  |
| **Adjuvant trastuzumab*** | 386 | 3.7 | 3429 | 21.46 | <.0001 | 335 | 3.55 | 2036 | 21.57 | <.0001 |
| **Comorbidity** |  |  |  |  |  |  |  |  |  |  |
| Diabetes mellitus | 1282 | 12.28 | 1459 | 9.13 | <.0001 | 977 | 10.35 | 911 | 9.65 | 0.1094 |
| Rheumatoid | 314 | 3.01 | 474 | 2.97 | 0.8409 | 277 | 2.93 | 272 | 2.88 | 0.8285 |
| Osteoporosis | 3308 | 31.7 | 3886 | 24.31 | <.0001 | 2887 | 30.59 | 2403 | 25.46 | <.0001 |
| COPD | 475 | 4.55 | 501 | 3.13 | <.0001 | 360 | 3.81 | 341 | 3.61 | 0.4646 |
| Depressive disorders | 709 | 6.79 | 1080 | 6.76 | 0.9089 | 607 | 6.43 | 648 | 6.87 | 0.231 |
| Anxiety disorders | 1078 | 10.33 | 1483 | 9.28 | 0.0048 | 924 | 9.79 | 917 | 9.72 | 0.8636 |
| Sleep disorder | 1389 | 13.31 | 2143 | 13.41 | 0.817 | 1200 | 12.71 | 1350 | 14.3 | 0.0014 |
| Hyperlipidemia | 3078 | 29.49 | 4078 | 25.52 | <.0001 | 2591 | 27.45 | 2529 | 26.79 | 0.3101 |
| Hypertension | 2377 | 22.78 | 2890 | 18.08 | <.0001 | 1809 | 19.17 | 1873 | 19.84 | 0.2398 |
| Other cardiovascular diseases | 486 | 4.66 | 818 | 5.12 | 0.0906 | 405 | 4.29 | 507 | 5.37 | 0.0005 |
| Renal failure | 68 | 0.65 | 74 | 0.46 | 0.0404 | 56 | 0.59 | 41 | 0.43 | 0.1268 |
| Chronic liver diseases | 1548 | 14.83 | 2182 | 13.65 | 0.0071 | 1330 | 14.09 | 1314 | 13.92 | 0.7372 |
| Cerebrovascular disease | 204 | 1.95 | 203 | 1.27 | <.0001 | 158 | 1.67 | 122 | 1.29 | 0.0302 |
| Anemia | 951 | 9.11 | 1606 | 10.05 | 0.0119 | 852 | 9.03 | 930 | 9.85 | 0.0522 |
| **Radiotherapy*** |  |  |  |  | <.0001 |  |  |  |  | <.0001 |
| Yes | 7824 | 74.97 | 12497 | 78.19 |  | 7258 | 76.89 | 7564 | 80.14 |  |
| **Number of sessions** |  |  |  |  | <.0001 |  |  |  |  | <.0001 |
| 0 | 2612 | 25.03 | 3485 | 21.81 |  | 2181 | 23.11 | 1875 | 19.86 |  |
| 1–10 | 1549 | 14.84 | 2090 | 13.08 |  | 1381 | 14.63 | 1381 | 14.63 |  |
| 11–20 | 486 | 4.66 | 868 | 5.43 |  | 426 | 4.51 | 426 | 4.51 |  |
| 21–30 | 2393 | 22.93 | 4089 | 25.59 |  | 2261 | 23.95 | 2342 | 24.81 |  |
| ≥31 | 3396 | 32.54 | 5450 | 34.1 |  | 3190 | 33.8 | 3415 | 36.18 |  |
| CCI: Charlson comorbidity index; COPD: chronic obstructive pulmonary disease | | | | | | | | | | |
