# Supplementary material for: Risk of cardiovascular disease and death in patients with breast cancer receiving anthracycline-based therapy: A retrospective cohort study
Source: PLoS One. 2025 Dec 4;20(12):e0335083. doi: 10.1371/journal.pone.0335083 (PMC12677787; doi:10.1371/journal.pone.0335083)
Supplement: S3 Table — (DOCX) [file pone.0335083.s003.docx]

**Supplementary Table S3. Frequency of adjuvant anticancer therapy administered within 1 month of the index date**

| **Adjuvant chemotherapy*** | **Before matching** | | | | |  | **After matching** | | | | |
| --- | --- | --- | --- | --- | --- | --- | --- | --- | --- | --- | --- |
|  | **Non-anthracycline (n = 10,436)** | | **Anthracycline (n = 15,982)** | | **P-value** |  | **Non-anthracycline (n = 9,439)** | | **Anthracycline (n = 9,439)** | | **P-value** |
|  | **N** | **%** | **N** | **%** |  |  | **N** | **%** | **N** | **%** |  |
| Epirubicin | - | - | 1487 | 9.3 | <.0001 |  | - | - | 907 | 9.61 | <.0001 |
| Taxane | 143 | 1.37 | 6475 | 40.51 | <.0001 |  | 126 | 1.33 | 3656 | 38.73 | <.0001 |
| SERM | 6332 | 60.67 | 7536 | 47.15 | <.0001 |  | 6051 | 64.11 | 4225 | 44.76 | <.0001 |
| Aromatase inhibitor | 3552 | 34.04 | 3763 | 23.55 | <.0001 |  | 2886 | 30.58 | 2361 | 25.01 | <.0001 |
| Methotrexate | 2125 | 20.36 | 31 | 0.19 | <.0001 |  | 1905 | 20.18 | 18 | 0.19 | <.0001 |
| 5-FU | 2120 | 20.31 | 3442 | 21.54 | 0.0172 |  | 1901 | 20.14 | 2095 | 22.2 | <.0001 |
| Dexrazoxane | - | - | 88 | 0.55 | - |  | - | - | 69 | 0.73 | - |
| *Followed up until 1-year follow-up | | |  |  |  |  |  |  |  |  |  |
