# Supplementary material for: Risk of cardiovascular disease and death in patients with breast cancer receiving anthracycline-based therapy: A retrospective cohort study
Source: PLoS One. 2025 Dec 4;20(12):e0335083. doi: 10.1371/journal.pone.0335083 (PMC12677787; doi:10.1371/journal.pone.0335083)
Supplement: S4 Table — (DOCX) [file pone.0335083.s004.docx]

**Supplementary Table S4. Multivariable Cox regression analysis for cardiovascular events in patients not treated with trastuzumab**

| **Covariates** | **Outcomes** | | | | | |
| --- | --- | --- | --- | --- | --- | --- |
|  | **Cardiovascular event*** | **Coronary artery disease and cardiac arrest** | **Congestive heart failure** | **Heart failure and cardiomyopathy** | **Stroke** | **All-cause mortality** |
|  | **aHR (95% CI)** | **aHR (95% CI)** | **aHR (95% CI)** | **aHR (95% CI)** | **aHR (95% CI)** | **aHR (95% CI)** |
| **Anthracycline (vs. No)** |  |  |  |  |  |  |
| Yes | 0.988 (0.894–1.091) | 0.985 (0.866–1.120) | 1.056 (0.892–1.251) | 1.055 (0.875–1.272) | 0.949 (0.766–1.177) | 2.335 (2.027–2.689) |
| **Age (vs. < 45)** |  |  |  |  |  |  |
| 45-54 | 1.447 (1.235–1.696) | 1.441 (1.173–1.771) | 1.385 (1.050–1.828) | 1.458 (1.071–1.986) | 1.454 (1.011–2.091) | 0.909 (0.759–1.089) |
| 55-64 | 2.295 (1.939–2.717) | 2.332 (1.876–2.899) | 2.129 (1.586–2.858) | 2.363 (1.705–3.273) | 2.639 (1.808–3.852) | 1.537 (1.263–1.871) |
| ≥65 | 3.188 (2.620–3.881) | 2.921 (2.261–3.773) | 3.504 (2.521–4.870) | 3.853 (2.672–5.555) | 5.056 (3.330–7.677) | 2.922 (2.322–3.677) |
| **CCI (vs. 2)** |  |  |  |  |  |  |
| 3-4 | 0.942 (0.806–1.101) | 0.901 (0.737–1.100) | 1.113 (0.853–1.451) | 1.082 (0.809–1.449) | 0.976 (0.706–1.349) | 0.882 (0.715–1.089) |
| ≥5 | 0.856 (0.744–0.986) | 0.790 (0.658–0.948) | 1.057 (0.832–1.342) | 1.056 (0.814–1.371) | 0.769 (0.569–1.038) | 0.995 (0.830–1.192) |
| **Income quintile (vs. 1st quintile)** |  |  |  |  |  |  |
| 2nd quintile | 0.801 (0.703–0.913) | 0.793 (0.672–0.936) | 0.856 (0.682–1.074) | 0.816 (0.635–1.049) | 0.695 (0.526–0.917) | 0.863 (0.726–1.026) |
| 3rd quintile | 1.349 (1.183–1.539) | 1.385 (1.168–1.642) | 1.243 (0.994–1.555) | 1.153 (0.904–1.471) | 1.365 (1.022–1.822) | 1.004 (0.853–1.181) |
| 4th quintile | 1.535 (1.305–1.806) | 1.543 (1.250–1.904) | 1.441 (1.095–1.897) | 1.385 (1.027–1.869) | 1.308 (0.908–1.884) | 1.343 (1.101–1.637) |
| **Comorbidity** |  |  |  |  |  |  |
| Diabetes mellitus | 1.260 (1.094–1.451) | 1.229 (1.025–1.473) | 1.232 (0.973–1.559) | 1.111 (0.851–1.451) | 1.367 (1.015–1.842) | 0.982 (0.800–1.206) |
| Rheumatoid | 1.213 (0.960–1.533) | 1.124 (0.826–1.530) | 1.166 (0.785–1.732) | 1.303 (0.855–1.986) | 1.797 (1.172–2.756) | 1.115 (0.807–1.541) |
| Osteoporosis | 1.006 (0.902–1.122) | 1.0  92 (0.950–1.254) | 1.023 (0.850–1.230) | 1.060 (0.866–1.298) | 0.902 (0.711–1.143) | 0.918 (0.792–1.064) |
| Chronic obstructive pulmonary disease | 0.934 (0.749–1.165) | 0.835 (0.619–1.125) | 1.079 (0.765–1.521) | 0.993 (0.671–1.471) | 0.853 (0.535–1.360) | 0.879 (0.643–1.201) |
| Depressive disorders | 1.145 (0.959–1.367) | 1.238 (0.990–1.548) | 1.214 (0.899–1.638) | 1.080 (0.770–1.514) | 0.966 (0.655–1.425) | 1.288 (1.020–1.626) |
| Anxiety disorders | 1.181 (1.019–1.368) | 1.184 (0.980–1.429) | 1.043 (0.806–1.349) | 1.047 (0.789–1.390) | 1.200 (0.878–1.640) | 1.023 (0.831–1.259) |
| Sleep disorder | 1.188 (1.039–1.359) | 1.134 (0.953–1.349) | 1.058 (0.838–1.336) | 1.237 (0.966–1.583) | 1.289 (0.975–1.703) | 1.010 (0.839–1.217) |
| Hyperlipidemia | 1.159 (1.032–1.303) | 1.322 (1.139–1.534) | 1.109 (0.911–1.351) | 1.088 (0.875–1.352) | 0.965 (0.749–1.242) | 0.995 (0.850–1.165) |
| Hypertension | 1.518 (1.346–1.712) | 1.453 (1.244–1.696) | 2.045 (1.672–2.502) | 1.863 (1.492–2.327) | 1.357 (1.046–1.759) | 1.189 (1.008–1.404) |
| Other cardiovascular diseases | 1.719 (1.455–2.030) | 1.741 (1.410–2.150) | 1.962 (1.515–2.541) | 2.021 (1.521–2.685) | 0.915 (0.594–1.409) | 1.234 (0.963–1.582) |
| Renal failure | 2.138 (1.454–3.144) | 2.165 (1.338–3.503) | 3.129 (1.880–5.208) | 3.560 (2.063–6.141) | 2.956 (1.426–6.130) | 3.258 (2.096–5.066) |
| Chronic liver diseases | 0.963 (0.841–1.103) | 0.953 (0.802–1.133) | 0.834 (0.659–1.056) | 0.832 (0.639–1.082) | 1.084 (0.812–1.447) | 1.111 (0.927–1.333) |
| Cerebrovascular disease | 1.699 (1.319–2.188) | 1.385 (0.983–1.951) | 0.876 (0.519–1.481) | 1.048 (0.607–1.808) | 3.650 (2.436–5.469) | 0.852 (0.530–1.370) |
| Anemia | 1.218 (1.040–1.425) | 1.188 (0.971–1.454) | 1.355 (1.049–1.751) | 1.289 (0.966–1.720) | 1.036 (0.719–1.492) | 1.263 (1.033–1.545) |
| **Radiotherapy (vs. 0)** |  |  |  |  |  |  |
| 1-10 | 1.007 (0.852–1.189) | 1.105 (0.890–1.373) | 1.041 (0.784–1.383) | 1.030 (0.751–1.412) | 0.881 (0.622–1.247) | 0.812 (0.661–0.998) |
| 11-20 | 1.118 (0.890–1.405) | 1.325 (0.995–1.764) | 1.220 (0.835–1.783) | 1.373 (0.918–2.054) | 0.634 (0.362–1.112) | 0.713 (0.522–0.975) |
| 21-30 | 1.036 (0.899–1.194) | 1.075 (0.891–1.296) | 1.072 (0.843–1.362) | 1.085 (0.832–1.414) | 0.882 (0.659–1.179) | 0.763 (0.638–0.912) |
| ≥31 | 0.953 (0.831–1.092) | 1.086 (0.909–1.298) | 0.969 (0.768–1.223) | 0.990 (0.765–1.280) | 0.717 (0.538–0.955) | 0.728 (0.613–0.864) |

aHR: adjusted hazard ratio; CCI: Charlson comorbidity index; CI: confidence interval;
