# Supplementary material for: Risk of cardiovascular disease and death in patients with breast cancer receiving anthracycline-based therapy: A retrospective cohort study
Source: PLoS One. 2025 Dec 4;20(12):e0335083. doi: 10.1371/journal.pone.0335083 (PMC12677787; doi:10.1371/journal.pone.0335083)
Supplement: S5 Table — (DOCX) [file pone.0335083.s005.docx]

**Supplementary Table S5. Multivariable Cox regression analysis for cardiovascular events in patients treated with trastuzumab**

| **Covariates** | **Outcomes** | | | | | |
| --- | --- | --- | --- | --- | --- | --- |
|  | **Cardiovascular event*** | **Coronary artery disease and cardiac arrest** | **Congestive heart failure** | **Heart failure and cardiomyopathy** | **Stroke** | **All–cause mortality** |
|  | **aHR (95% CI)** | **aHR (95% CI)** | **aHR (95% CI)** | **aHR (95% CI)** | **aHR (95% CI)** | **aHR (95% CI)** |
| **Anthracycline (vs. No)** |  |  |  |  |  |  |
| Yes | 1.042 (0.664–1.633) | 1.554 (0.809–2.984) | 0.939 (0.461–1.909) | 0.904 (0.426–1.919) | 0.725 (0.289–1.818) | 0.693 (0.386–1.245) |
| **Age (vs. < 45)** |  |  |  |  |  |  |
| 45–54 | 3.020 (1.158–7.873) | 3720169.86 (0.000–.) | 1.688 (0.534–5.332) | 1.399 (0.432–4.533) | 3.342 (0.401–27.823) | 0.395 (0.181–0.861) |
| 55–64 | 4.117 (1.551–10.928) | 5501906.24 (0.000–.) | 1.819 (0.545–6.069) | 1.693 (0.500–5.734) | 4.272 (0.484–37.728) | 0.820 (0.384–1.751) |
| ≥65 | 7.811 (2.865–21.299) | 10467579.5 (0.000–.) | 4.273 (1.215–15.034) | 3.666 (1.018–13.200) | 10.503 (1.183–93.215) | 1.747 (0.766–3.985) |
| **CCI (vs. 2)** |  |  |  |  |  |  |
| 3–4 | 1.103 (0.582–2.090) | 0.952 (0.358–2.528) | 1.534 (0.564–4.173) | 1.073 (0.369–3.120) | 0.890 (0.246–3.212) | 0.703 (0.293–1.687) |
| ≥5 | 0.608 (0.343–1.078) | 0.611 (0.277–1.351) | 0.822 (0.326–2.075) | 0.709 (0.270–1.861) | 0.471 (0.152–1.463) | 0.801 (0.391–1.644) |
| **Income quintile (vs. 1st quintile)** |  |  |  |  |  |  |
| 2nd quintile | 0.850 (0.497–1.454) | 0.857 (0.406–1.807) | 0.818 (0.328–2.039) | 0.740 (0.291–1.884) | 0.694 (0.248–1.946) | 0.857 (0.426–1.723) |
| 3rd quintile | 1.777 (1.054–2.994) | 1.565 (0.738–3.320) | 2.262 (1.028–4.979) | 1.976 (0.877–4.451) | 1.341 (0.480–3.747) | 1.275 (0.680–2.391) |
| 4th quintile | 1.465 (0.734–2.926) | 1.190 (0.448–3.159) | 1.352 (0.403–4.532) | 1.251 (0.348–4.505) | 1.136 (0.292–4.422) | 1.866 (0.830–4.196) |
| **Comorbidity** |  |  |  |  |  |  |
| Diabetes mellitus | 0.953 (0.532–1.709) | 1.296 (0.587–2.860) | 0.371 (0.104–1.328) | 0.290 (0.063–1.323) | 1.185 (0.397–3.539) | 1.062 (0.455–2.478) |
| Rheumatoid | 0.463 (0.110–1.954) | 0.494 (0.065–3.759) | 1.611 (0.355–7.306) | 1.035 (0.131–8.150) | 0.000 (0.000–.) | 0.000 (0.000–.) |
| Osteoporosis | 0.936 (0.600–1.459) | 1.417 (0.786–2.555) | 0.628 (0.296–1.332) | 0.751 (0.339–1.662) | 0.782 (0.315–1.939) | 0.421 (0.205–0.864) |
| Chronic obstructive pulmonary disease | 0.598 (0.233–1.532) | 0.623 (0.182–2.127) | 0.977 (0.276–3.457) | 1.256 (0.349–4.526) | 1.060 (0.218–5.160) | 0.416 (0.095–1.819) |
| Depressive disorders | 1.158 (0.548–2.446) | 1.490 (0.549–4.044) | 0.782 (0.211–2.898) | 0.257 (0.031–2.103) | 0.404 (0.051–3.203) | 0.667 (0.199–2.234) |
| Anxiety disorders | 1.067 (0.581–1.961) | 1.212 (0.521–2.818) | 0.852 (0.304–2.388) | 0.649 (0.183–2.304) | 0.783 (0.216–2.842) | 0.932 (0.349–2.486) |
| Sleep disorder | 1.635 (0.997–2.680) | 0.830 (0.383–1.801) | 1.898 (0.879–4.100) | 1.865 (0.794–4.379) | 2.525 (1.006–6.340) | 1.140 (0.541–2.405) |
| Hyperlipidemia | 1.274 (0.819–1.983) | 0.798 (0.417–1.526) | 1.508 (0.742–3.062) | 1.038 (0.469–2.300) | 1.599 (0.676–3.783) | 0.718 (0.365–1.411) |
| Hypertension | 1.453 (0.919–2.297) | 1.672 (0.883–3.165) | 1.256 (0.587–2.690) | 1.471 (0.655–3.302) | 1.447 (0.575–3.642) | 0.825 (0.409–1.663) |
| Other cardiovascular diseases | 0.429 (0.153–1.198) | 0.634 (0.189–2.126) | 0.824 (0.220–3.089) | 0.477 (0.088–2.573) | 0.369 (0.047–2.916) | 1.246 (0.434–3.579) |
| Renal failure | 1.031 (0.122–8.746) | 0.000 (0.000–.) | 14.833 (1.029–213.904) | 27.757 (1.470–524.247) | 0.000 (0.000–.) | 0.000 (0.000–.) |
| Chronic liver diseases | 0.668 (0.376–1.186) | 1.048 (0.480–2.285) | 0.425 (0.141–1.281) | 0.570 (0.186–1.751) | 0.386 (0.112–1.337) | 0.697 (0.300–1.617) |
| Cerebrovascular disease | 0.939 (0.270–3.263) | 0.716 (0.089–5.783) | 0.000 (0.000–.) | 0.000 (0.000–.) | 2.514 (0.412–15.340) | 0.000 (0.000–.) |
| Anemia | 1.434 (0.710–2.896) | 1.412 (0.523–3.813) | 0.283 (0.037–2.176) | 0.288 (0.036–2.316) | 4.024 (1.297–12.485) | 1.846 (0.794–4.295) |
| **Radiotherapy (vs. 0)** |  |  |  |  |  |  |
| 1–10 | 1.211 (0.601–2.441) | 1.133 (0.412–3.117) | 1.183 (0.386–3.630) | 1.297 (0.393–4.284) | 1.222 (0.309–4.829) | 0.729 (0.264–2.009) |
| 11–20 | 0.000 (0.000–.) | 0.000 (0.000–.) | 0.000 (0.000–.) | 0.000 (0.000–.) | 0.000 (0.000–.) | 1.862 (0.230–15.067) |
| 21–30 | 0.927 (0.504–1.704) | 0.941 (0.404–2.195) | 0.856 (0.307–2.390) | 0.751 (0.242–2.332) | 1.037 (0.325–3.302) | 0.931 (0.428–2.025) |
| ≥31 | 1.031 (0.576–1.843) | 0.954 (0.419–2.172) | 1.097 (0.430–2.802) | 1.166 (0.434–3.134) | 1.088 (0.346–3.418) | 1.001 (0.489–2.051) |

aHR: adjusted hazard ratio; CCI: Charlson comorbidity index; CI: confidence interval;
